# The evolution of novel fungal genes from non-retroviral RNA viruses

**DOI:** 10.1186/1741-7007-7-88

**Published:** 2009-12-18

**Authors:** Derek J Taylor, Jeremy Bruenn

**Affiliations:** 1 Department of Biological Sciences, The State University of New York at Buffalo, Buffalo, NY 14260, USA

## Abstract

**Background:**

Endogenous derivatives of non-retroviral RNA viruses are thought to be absent or rare in eukaryotic genomes because integration of RNA viruses in host genomes is impossible without reverse transcription. However, such derivatives have been proposed for animals, plants and fungi, often based on surrogate bioinformatic evidence. At present, there is little known of the evolution and function of integrated non-retroviral RNA virus genes. Here, we provide direct evidence of integration by sequencing across host-virus gene boundaries and carry out phylogenetic analyses of fungal hosts and totivirids (dsRNA viruses of fungi and protozoans). Further, we examine functionality by tests of neutral evolution, comparison of residues that are necessary for viral capsid functioning and assays for transcripts, dsRNA and viral particles.

**Results:**

Sequencing evidence from gene boundaries was consistent with integration. We detected previously unknown integrated *Totivirus*-like sequences in three fungi (*Candida parapsilosis*, *Penicillium marneffei *and *Uromyces appendiculatus*). The phylogenetic evidence strongly indicated that the direction of transfer was from *Totivirus *to fungus. However, there was evidence of transfer of *Totivirus*-like sequences among fungi. Tests of selection indicated that integrated genes are maintained by purifying selection. Transcripts were apparent for some gene copies, but, in most cases, the endogenous sequences lacked the residues necessary for normal viral functioning.

**Conclusions:**

Our findings reveal that horizontal gene transfer can result in novel gene formation in eukaryotes despite miniaturized genomic targets and a need for co-option of reverse transcriptase.

## Background

In eukaryotes, novel genes can be formed by alternative splicing, exon shuffling, horizontal gene transfer and inserted retroelements [1,2]. Indeed, many eukaryotic genomes are bloated with the raw materials (introns and retroelements) for these processes [3-5]. In most budding yeasts, however, the source of novel gene formation is obscure as there is a dearth of spliceosomal introns (< 5% of genes and < 0.5% of the genome) and retroelement products (< 3% of genome size) [6-8]. Although duplication of existing genes is common in budding yeasts, *de novo *gene formation and the horizontal gene transfer (HGT) appear stifled by the architecture of miniaturized genomes [7,9]. It is surprising, then, that NCBI Genbank annotations (EU380679 from this study, CAR65487, ABN68085, and ABN68086) and the BLAST-based study of Frank and Wolfe [10] reveal significant matches of yeast genes to the non-retroviral dsRNA viruses *Saccharomyces cerevisiae *L-A (L1) virus (Totiviridae). *Debaryomyces hansenii *has two capsid (Cp)-like genes, while *Pichia stipitis *has at least four Cp-like genes; each fungus has a single RNA dependent RNA polymerase (RdRp)-like gene [10]. Endogeny is common for viruses that either encode their own reverse transcriptase (retroviruses and pararetroviruses) or are already DNA-based [11-13] but fragments of integrated non-retroviral viral RNA have rarely been proposed [14-16]. As the integration of non-retroviral RNA viruses into DNA-based eukaryotic genomes requires the co-option of reverse transcriptase [17], Holmes [12] called this type of transfer 'one of the most remarkable observations in viral evolution of recent years'.

Little is known of the biology of non-retroviral integrated RNA viruses (NIRVs). We are unaware, for example, of evolutionary or functional comparisons among NIRVs. Even the initial bioinformatic evidence of NIRVs is often weak as genome assemblies can be contaminated or incorrectly annotated and surrogate (non-phylogenetic) analyses are susceptible to false positives [18-20]. The proposed transfer of bacterial genes into the human genome, for example, disappeared with a detailed phylogenetic analysis [21]. The initial claims of NIRVs in grape (*Vitus*) genomes also failed the direct tests of integration [22]. Direct evidence of NIRVs is provided by the successful polymerase chain reaction (PCR) amplification or sequencing across host and integrated virus gene boundaries [14]. Where HGT is strongly supported, BLAST-based analyses of genes with open reading frames cannot adequately discern the direction of the transfer (from host to virus or from virus to host). The dsRNA elements that code for killer toxins in some fungi appear to have a cellular origin based on structural similarities to cellular genes and, in some cases, the preservation of vestigial polyA sequences at internal positions of the viral plus strands [23]. However, another BLAST-based study concluded that significant sequence matches with cellular genes and the presence of cellular pseudogenes indicated the transfer from killer dsRNA element to cellular genome [10]. The resolution of the question of directionality and the determination of HGT requires phylogenetic evidence with strong support and adequate sampling of interacting organisms [18].

Nor can functional maintenance of suspected HGT genes be inferred solely from a putative open reading frame - recent pseudogenes can produce a similar pattern [24]. Evolutionary analysis of selection and analysis of transcription products can provide stronger evidence of functional maintenance. Though negative evidence, a lack of viral products (dsRNA or viral particles), might hint at a novel function in a NIRV. Further evidence can be provided by the comparative analysis of functional landmarks. For example, the capsid gene of the *Totivirus *(L1-LA) functions to remove m^7^GMP caps from fungal host mRNAs [25,26]. Site directed mutational experiments have revealed eight residues that are essential for this decapping function [25,26]. Alteration of these residues in an integrated viral Cp gene would provide evidence for novel function. In the present study we carry out phylogenetic, evolutionary and functional analyses to test the hypothesis of NIRVs, the direction of HGT and the hypothesis that HGT of non-retroviral RNA viruses results in novel functioning genes in the fungal-Totiviridae system.

Totivirids are simple dsRNA viruses defined by the presence of the RdRp and the single capsid polypeptide (Cp) on a single dsRNA [27]. The isometric virions are about 40 nm in diameter. Inheritance of these intracellular RNA viruses is normally vertical (no cellular genomic copies of the dsRNA genomes are present)[28] but horizontal transfer can occur across fungal hyphae [27]. Confirmed intracellular totivirid infections are known only from fungi and pathogenic protozoans (*Leishmania*, *Giardia *and *Trichonomas*). The totivirids in smut and yeast have been assigned to the genus *Totivirus*, whereas totivirids infecting filamentous fungi have been assigned to the genus *Victorivirus *[29]. Here, we provide independent direct evidence that *Totivirus*-like genomes are integrated into the cellular genomes of yeast that lack *Totivirus *infections and phylogenetic evidence that genes of *Totivirus*-like viruses have been transferred to the genomes of four yeast species. Moreover, the endogenous *Totivirus*-like genes appear to be functionally maintained with non-viral functions and may have been further transferred between deeper yeast lineages.

## Results and discussion

**Figure 1:**
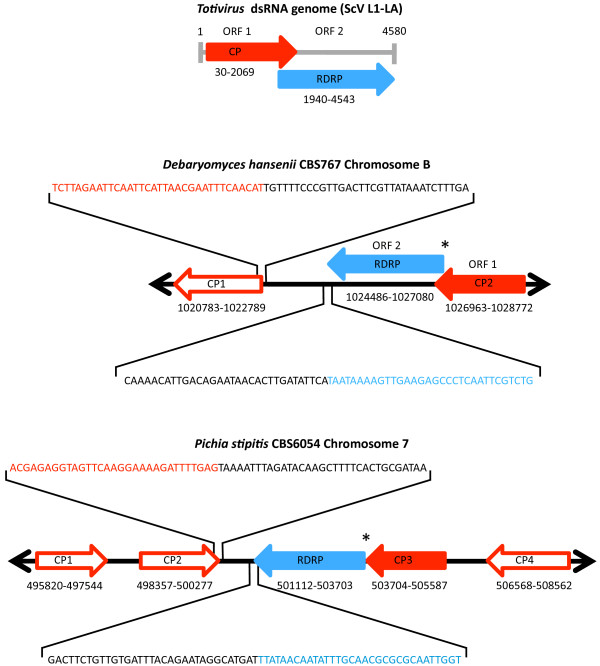
**A comparison of the simple genome of *Totivirus*, a dsRNA virus, with the fungal genome maps containing proposed endogenous *Totivirus*-like genes and open reading frames**. Red arrows indicate capsid polypeptide-like genes and the blue arrows represent RNA-dependent RNA polymerase-like genes. These coloured arrows represent open reading frames). Gray lines represent an RNA based genome and black lines represent fungal chromosomal DNA. Gene copies that retain an exogenous viral genomic architecture are shown as solid coloured arrows with the coding strand indicated by the direction of the arrow. Sequences from the present study that reveal fungal-*Totivirus*-like genes boundaries are shown above the maps. Asterisks represent further gene boundary regions amplified successfully by polymerase chain reaction (PCR; see Figure 6A). Numbers below the gene annotations indicate the chromosomal positions of gene boundaries. Full sequences of the PCR products from these regions have the Genbank Accession numbers: GQ291318-GQ291321.

**Figure 2:**
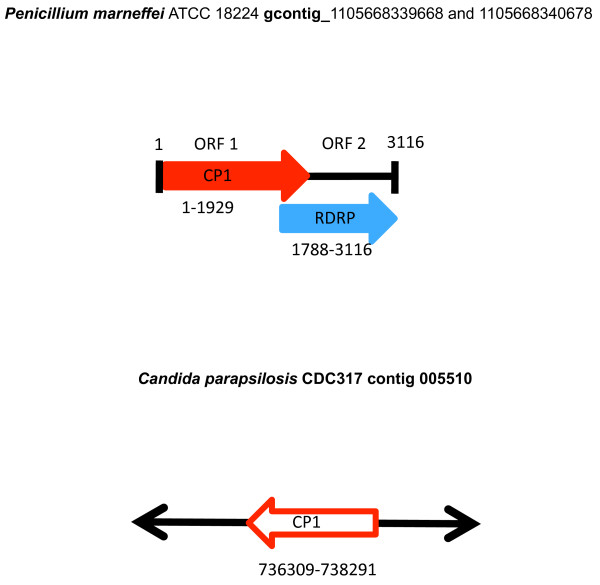
**A comparison of the fungal genome maps of *Penicillium *and *Candida *proposed to contain endogenous *Totivirus*-like genes**. Red arrows indicate capsid polypeptide-like genes and the blue arrows represent RNA-dependent RNA polymerase-like genes. These coloured arrows represent open reading frames. Black lines represent fungal chromosomal DNA. Gene copies that retain an exogenous viral genomic architecture are shown as solid coloured arrows with the coding strand indicated by the direction of the arrow. Numbers below the gene annotations indicate the contig positions of gene boundaries.

BLAST analysis using the RdRp and Cp protein sequences of the *S. cerevisiae *virus La (L-Bc) as a query, detected *Totivirus*-like genes in five fungal species. We found RdRp-like sequences in *P. stipitis*, *Penicillium marneffei*, *D. hansenii *and *Uromyces appendiculatus*, and Cp-like sequences in *P. stipitis*, *P. marneffei*, *D. hansenii*, and *Candida parapsilosis*. The *U. appendiculatus *match is from an Expressed Sequence Tags (EST) database. The *D. hansenii *virus-like genome has two overlapping reading frames, as in the exogenous viral architecture of the *S. cerevisiae *L1 virus (ScVL1 or ScVL-A; Figure 1; [10]. The overlap is 138 bases and the RdRp-like gene can be translated as a minus one (-1) programmed ribosomal frameshift with the slippery site, GGGUUUA [30,31], as in ScVL1. The *P. stipitis *virus-like genes encode a Cp-RdRp fusion protein, and has a similar architecture to that found in the *Ustilago maydis *P1H1 virus UmV [32]. The *P. marneffei *genome also appears to contain a *Totivirus*-like genome (Figure 2). Here the overlap between the CP-like gene and the RdRp-like gene is 141 bases, but the slippery site that would allow a minus one translation has been altered by a point mutation (GGAUUUA), suggesting a non-viral function. No nonviral-like sequence is available from existing contigs of *P. marneffei*, so further sequencing would be needed in order to confirm the fungal flanking regions. For *C. parapsilosis*, it is clear that only a Cp-like sequence is present on the large contig where we detected a *Totivirus*-like gene (Figure 2).

We tested for the presence of DNA copies of dsRNA *Totivirus*-like genes by PCR amplification of fungal DNA extractions with specific primers (Figure 1). The first assay, targeting the RdRp-Cp boundary, revealed an expected PCR product size from both *P. stipitis *(488 bp) and *D. hansenii *(1172 bp), while *S. cerevisiae*, which possesses only viral dsRNA targets, lacked a detectable PCR product. This result suggests that DNA-based genes matching the viral sequences are present in *D. hansenii *and *P. stipitis *cells. We then tested for fungal integration of these genes by PCR amplification and DNA sequencing across the proposed fungal-viral genome boundaries. We sequenced the RdRp-fungus and upstream Cp-fungus boundaries for each fungal species (Figure 1). The experimental sequences have 100% matches with the proposed yeast genome assemblies containing both the expected yeast and the viral-like sequences. A direct test of the integration in the remaining three fungi where we detected *Totivirus*-like sequences is pending.

**Figure 3:**
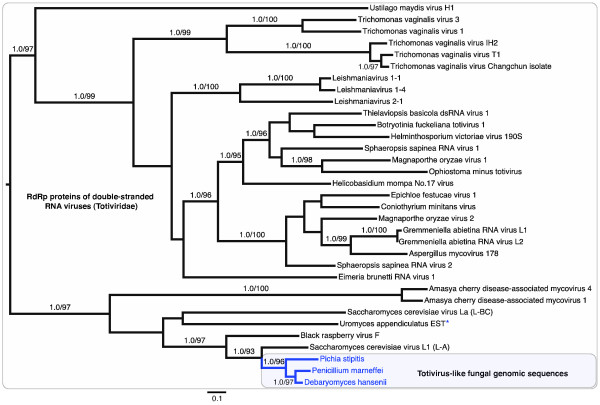
**Maximum likelihood phylograms of totivirid-like RNA-dependent RNA polymerase genes based on an alignment of amino acid sequences**. Bayesian posterior probability and nonparametric bootstrap values >85% are figured above branches. Fungal genomic sequences are highlighted by a blue box. The asterisk represents a putative fungal expressed sequence tag sequence.

**Figure 4:**
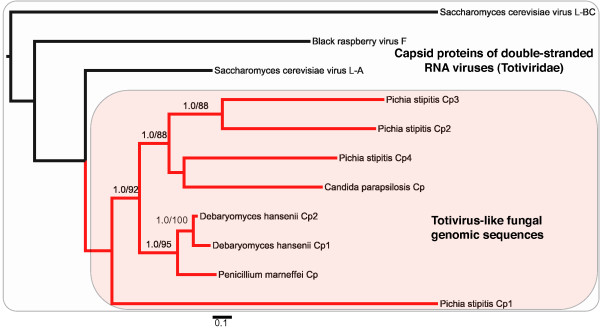
**Maximum likelihood phylogram of totivirid-like capsid polypeptide (Cp)-like genes based on an alignment of amino acid sequences**. Bayesian posterior probability and nonparametric bootstrap values > 85% are figured above branches. Fungal genomic sequences are highlighted with a red box. Within-species copies are numbered as in Figure 1.

The fungal RdRp-like sequences form a close, and strongly supported, derived group within the viral RdRp gene tree (Figure 3). Thus, the data satisfy the phylogenetic criterion of horizontal transfer - a strongly supported phylogenetic incongruence between interacting organisms [18]. As many fungal genomes are now known, the close association of the viral-like RdRp gene in hemiascomycetous yeast (*P. stipitis *and *D. hansenii*) with the RdRp gene of exogenous *Totivirus *of other hemiascomycetes, such as *S. cerevisiae *(Figures 3-4), is unlikely to be a sampling artifact or a differential gene loss of *Totivirus*-like genes in fungi. Instead, the placement of virus-like fungal sequences at the tips of the totivirid tree indicates that the endogenous forms in hemiascomycetes evolved from *Totivirus *and not from fungal genomes. The *Totivirus*-like genes of *P. marneffei*, a Euascomycote (Additional File 1), are also nested within the hemiascomycete clade and are most closely related to *D. hansenii*. The presence of closely related *Totivirus *RdRp-like sequences in fungi from two divergent clades suggests the occurrence of multiple integration events or horizontal transfers among yeasts. The position of the fungal sequences within the hemiascomycete virus clade suggests that integration occurred first in hemiascomycetes and was transferred to *P. marneffei*. Further evidence of homology of NIRVs might be provided by similarity of chromosomal regions flanking the insertion site. However, even the most closely related fungi in our study have undergone extensive chromosomal rearrangements. Jeffries *et al*. [8] found no orthologous chromosomal segments between the center of CHR 7 in *P. stipitis *and chromosome B of *D. hansenii*, the locations of the NIRVs. The fungal gene tree (Additional File 1) reveals that the fungal species with endogenous *Totivirus*-like genes, save *P. marneffei*, belong to the Clavispora clade [33], which has *Candida lusitaniae *as the representative species. The Clavispora clade, which has sometimes been called the *Candida *clade [9] and is characterized by a shift in genetic code, is estimated to be at least 100 million years old [33].

The Cp gene tree also revealed a close relationship of the endogenous viruses to the *Totivirus *of Hemiascomycotes (Figure 4). BLAST matches are apparently limited to the genus *Totivirus *because the Cp gene has evolved more rapidly than the RdRp gene (Figures 3-4). The two copies of Cp-like genes within *D. hansenii *have a sister relationship (amino-acid *p*-distance = 0.060) and represent either recent paralogous duplication or concerted evolution. However, the four copies of Cp-like genes in *P. stipitis *are much more divergent with the average amino *p*-distance between Cp1 and the other Cp copies in *Pichia *at 0.755. The tandem positioning of two to four divergent Cp gene copies and the monophyly of the fungal viral-like genes is consistent with the hypothesis of endogenous tandem duplication after integration by a *Totivirus*-like dsRNA viral lineage. Interestingly, the ancestral viral genomic architecture appears intact in *P. stipitis *(similar to UmV, see above)*D. hansenii *(similar to ScVL1) and *P. marneffei *which further supports the viral genome transfer hypothesis and permits the diagnosis of the ancestral integrated gene in the Cp-like gene family (Figures 1-2). Alternative scenarios, where *Totivirus *genomes are repeatedly and faithfully duplicated *in toto *or are independently integrated at the identical regions in these fungal genomes, are unlikely.

Under the scenario of endogeny we expect patterns of DNA substitutions to differ between exogenous viral and integrated fungal gene copies. A disparity index revealed that fungal sequences do differ from viral sequences in patterns of DNA substitution more than is expected from evolutionary distance or from chance alone (Additional File 2). Apart from the Cp1 sequence in *P. stipitis*, there were no significant differences among disparity indices within the fungal sequences or within the viral sequences. Thus, our sequencing of gene boundaries and the substitution patterns are consistent with integrated fungal copies of dsRNA *Totivirus*-like genes.

**Figure 5:**
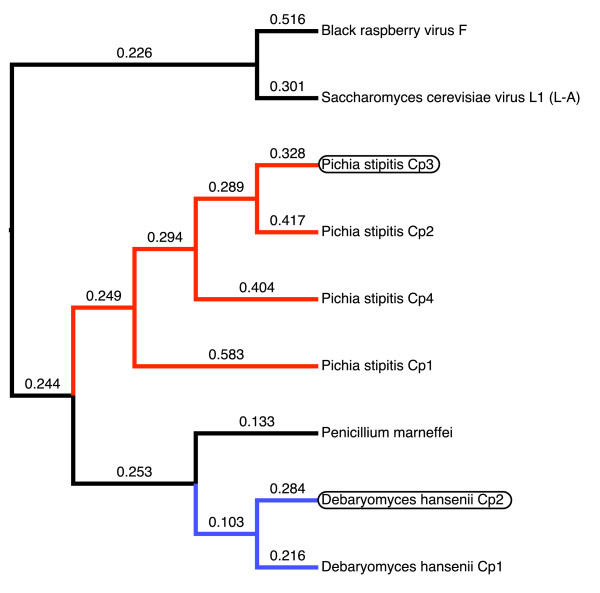
**Binary rooted tree of capsid protein-like nucleotide sequences with Ka/Ks ratios plotted on branches**. The tree is constrained with the assumption of tandem array monophyly. The relationships of gene family members are shown for *Pichia stipitis *(in red) and for *Debaryomyces hansenii *(in blue). Genes associated with the ancestral architecture are highlighted with a black oval.

Despite pronounced sequence divergence, each of the *Totivirus*-like fungal genes had an uninterrupted open reading frame (Figures 1-2). By searching the translated EST database for *Pichia *with the RdRp amino acid sequence (tBLASTn), we found that the RdRp mRNA product appears as a normal, polyadenylated RNAPII transcript in EST libraries (FE843929.1 and FE843928.1). Similarly, we found matching RNAPII transcripts of at least two of the Cp genes, Cp2 (FE851263.1 and FE851264.1) and Cp4 (FE849285.1 and FE849284.1) in *P. stipitis*. In order to test whether the endogenous genes are evolving as functional genes, we calculated pairwise and phylogenetic tests of neutral evolution (Table 1; Figure 5). Each of the comparisons for endogenous fungal genes revealed a significant departure from neutrality in the direction of purifying selection, consistent with purifying selection to maintain gene function (Table 1; Figure 5).

**Table 1:** A codon based test for neutral evolution in the capsid protein-like genes of *Totivirus *and nonretroviral integrated RNA viruses from fungi.

	1	2	3	4	5	6	7	8	9
1									
2	0.520								
3	-2.672	-2.440							
4	-3.995	-2.139	-5.286						
5	-4.294	-2.058	-7.457	-7.629					
6	-2.155	-2.696	-4.855	-4.261	-5.158				
7	-3.378	-2.240	-3.593	-2.973	-3.250	-2.983			
8	-2.160	-2.807	-2.807	-2.215	-2.125	-2.051	-3.806		
9	-2.091	-2.241	-2.087	-2.093	-2.800	-2.711	-2.306	-2.372	

1. *Saccharomyces cerevisiae *virus La (L-BC)

2. Black raspberry virus F

3. *Debaryomyces hansenii *Cp1

4. *D. hansenii *Cp2

5. *Penicillium marneffei*

6. *Pichia stipitis *Cp4

7. *P. stipitis *Cp2

8. *P. stipitis *Cp3

9. *P. stipitis *Cp1

The test statistic (*d*N - *d*S) is shown where *d*S and *d*N are the numbers of synonymous and nonsynonymous substitutions per site. The variance of the difference was computed using the bootstrap method (500 replicates). Analyses were conducted using the Kumar method in MEGA4. All negative values have a *P *value of less than 0.05 and are considered significant. The negative values indicate rejection of the hypothesis of strict neutrality in the direction of purifying selection (a negative *d*N-*d*S). Significance for the viral contrast (1 versus 2) could not be calculated because the evolutionary distance could not be calculated. The key to the cell numbers is given below the table.

**Figure 6:**
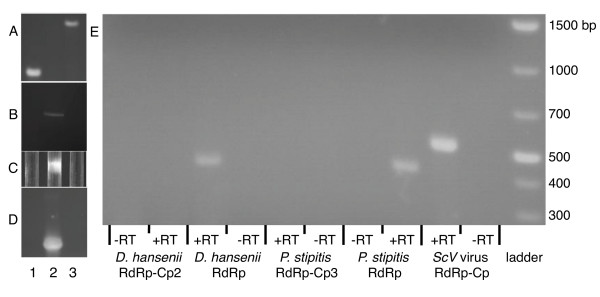
**A comparison of *Totivirus *and endogenous *Totivirus*-like fungal gene products**. *Pichia stipitis *(lane 1), *Saccharomyces cerevisiae *with exogenous *Totivirus *infection (lane 2) and *Debaryomyces hansenii *(lane 3) (A) polymerase chain reaction (PCR) of total DNA (1.4% agarose gel). (B) Assay for dsRNA (1.4% agarose gel with EtBr staining). (C) Assay for viral particles (photograph of CsCl density gradients). (D) Reverse transcriptase (RT)-PCR of total RNA (1.4% agarose gel). (E) PCR of total RNA extractions treated with DNAse. Here, RT-PCR of Cp is attempted from primers that flank the Cp-RdRp boundary. Reverse transcriptase is marked as either present (+RT) or absent (-RT).

**Figure 7:**
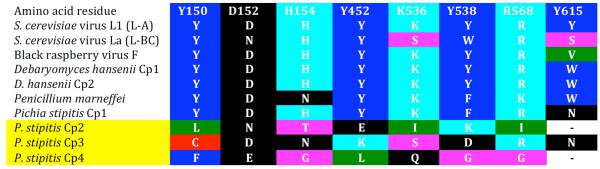
**A matrix showing conservation patterns in capsid protein-like genes at residues in *Saccharomyces cerevisiae *virus L1 (L-A) capsid protein that are structurally important for the decapping function**. Shading represents the seven amino acid categories assigned by size and charge in the GDE (Genetic Data Environment) format. Dashes are indels. Yellow-shaded names indicate fungal capsid-protein-like genes that lack conservation of functional residues for decapping (see Figure 1).

As we found evidence for viral genome architecture in our PCRs (Figures 1 and 6A), we examined if the endogenous genes functioned as in dsRNA viruses. We failed to detect dsRNA products (Figure 6B) or viral particles (empty or full) in *P. stipitis *and in *D. hansenii *(Figure 6C). This contrasts with the positive detection of dsRNA and viral particles in the *Totivirus *containing cells of *S. cerevisiae*. A reverse transcriptase PCR (RTPCR) experiment revealed no detectable transcripts of the complete endogenous *Totivirus *genomes in *D. hansenii *or in *P. stipitis *(targets include the Cp-RdRp boundary, Figure 6D). Notably, RTPCR with oligonucleotide primers targeting only the RdRp region did contain transcripts (Figure 6E). RNA from the *Totivirus*-containing *S. crevisiae *does give an RTPCR product from the Cp-RdRp region. These results indicate that endogenous gene transcription proceeds differently than in *Totivirus*, as the integrated RdRp sequences in *D. hansenii *and in *P. stipitis *initiate within the Cp sequence, creating a subgenomic mRNA. Further, six to seven of eight biochemically-conserved residues that are important to the decapping function for *Totivirus *Cp genes [25,26] are altered in three of the endogenous Cp-like genes of *P. stipitis *(Figure 7).

Taken together, the functional and comparative evidence suggests that the endogenous viral proteins have been co-opted for cellular functions. The abandonment of viral expression is also indicated by the absence of recognizable RNA pseudoknot structure, following the slippery site in the *Totivirus*-like genomes of *Pichia and P. marneffei*, and by the absence of recognizable proteinase cleavage sites or proteinase active site motifs in the Cp-RdRp overlap region of *D. hansenii*. Co-option of integrated DNA viruses and retroviruses by multicellular eukaryotes is well known but rare [34]. An integration mechanism that co-opts endogenous RNA integration machinery is plausible as yeast genomes contain retroelements [9,35]. However, the signature of retroelements is not obvious in the flanking regions of the present integrations, perhaps as a result of evolutionary divergence.

The evolutionary distribution and stabilization of NIRVs is poorly understood. Opportunities for NIRV formation should be greater in host taxa that have evolved a longstanding (presumably hypovirulent) non-retroviral virus infection, as in the fungi-*Totivirus *or the dipteran-flavivirus associations. However, the *Clavispora *clade, which we identify as the source of the yeast NIRVs, appears to lack such infections with *Totivirus*. Given the widespread taxonomic distribution of *Totivirus*-like infections in fungi and their absence in *Clavispora *[29], it is tempting to invoke the shift in genetic code as a contributor to the loss of infectionby *Totivirus*. Holmes [12] proposed that shifts in genetic codes are evolutionary responses by the host to RNA-based viral infections. NIRVs could have initially played a beneficial role to the host by imparting resistance to exogenous viruses. Indeed, there is experimental evidence in the yeast-*Totivirus *system that overproduction of the capsid protein [36] or production of fragments of the capsid protein[37] will interfere with packaging of the virus and result in its loss. Such interference is also well-documented with plant viruses [38]. The apparent genomic prevalence of capsid-like NIRVs compared to RdRp-like NIRVs (Figures 1 and 2) is consistent with this interference hypothesis. In either case, the NIRVs that we find in the *Clavispora *clade today could represent the vestiges of a co-evolutionary victory by the host.

## Conclusions

We conclude that novel eukaryotic gene families have originated from non-retroviral RNA viruses. NIRVs and their transitional stages are archived in eukaryotic genomes and appear more important to yeast evolution than previously thought.

## Methods

### Cell cultures

We obtained lyophilized powders of *P. stipitis *strain CBS6054 [8] from T Jeffries, *Debaryomyces hansenii *strain CBS767 [6] from Jean-Luc Souciet and *Saccharomyces cerevisiae *strain S7 from C McLaughlin [39], which has the *S. cerevisiae *virus L1 (LA) and the minor virus La (LB-C) but no satellite virus. Cells were streaked on YPD agar (yeast extract 1%, peptone 2%, and dextrose 2%) and single colonies were transferred to 150 ml of YPD broth.

### Nucleic acid and viral particle extractions

RNA was extracted from cells with the Masterpure Yeast RNA extraction kit (Epicentre, WI, USA). DNA was extracted using a standard SDS/phenol/chloroform protocol. Viral dsRNA was isolated by CF11 chromatography from crude RNA preparations [40]. Empty viral particles of density 1.33 g/cc were isolated as described [41], except that polyethylene glycol precipitation was replaced by high-speed pelleting of viral particles (100,000 × *g *for 1.5 h).

### PCR, RTPCR and DNA sequencing

Fifty microlitre PCR reactions contained 5 μL of extracted DNA template, 10× PCR buffer [50 mM KCl, 1.5 mg MgCl2, 10 mM Tris-HCl pH 8.3, 0.01% (w/v) gelatin], 2 mM of each dNTP, 1 μM of each primer and 1 unit of Taq DNA polymerase. Primers are listed in Additional File 3. The PCR temperature profiles were: 30 cycles of 94°C for 30 s; 55°C for 30 s; 72°C for 2 min; and final extension at 72°C for 5 min. RT-PCR detection of viral transcripts and PCR of genomic DNA copies (Epicentre high fidelity RT-PCR kit) were performed as described by the manufacturer. Gels were 1.4% agarose in Tris-acetate-EDTA (40 mM Tris-acetate and 1 mM EDTA, pH 8.3) stained with ethidium bromide (1 mg/l). DNA was sequenced by Sanger methods at the University of Washington High Throughput Genomics Facility. Sequencher 4.8 was used to assemble and edit electrophoregrams. New sequences from this study have the following Genbank accession numbers: EU380679 and GQ291318-GQ291321.

### Bioinformatics

We obtained the initial sequences of RdRp (Additional File 4) and capsid (Additional File 5) genes from totivirids by BLASTp of the nr peptide sequence database (National Center for Biotechnology Information, Bethesda, USA) with the protein sequences of *S. cerevisiae *virus La (L-BC) and a cut-off of E<0.01. Additional genomic copies of *Totivirus*-like genes were identified by significant tBLASTx hits (*E*<0.05) of relevant NCBI BLAST databases using the sequences of *S. cerevisiae *virus La (L-BC). For the capsid proteins the entire sequences were used and, for RdRp, the contiguous conserved region [27] was used. Sequences were aligned using Prank: probabilistic alignment kit with the Pranskter graphical interface (Goldman Group, European Bioinformatics Institute, Cambridge, United Kingdom) [42]. We carried out maximum likelihood analyses with RAxML using the RTREV substitution matrix, estimated AA frequencies, a gamma parameter for among-site rate variation, and an invariable sites parameter [43]. For bootstrapping, RAxML estimated the number of pseudoreplicates. For Bayesian analysis, we used Mr Bayes [44] with an amino acid substitution model prior of RTREV with a setting of rates = INVGAMMA. After 1 million Markov chain Monte Carlo generations and confirming convergence (average standard deviation of split frequencies < 0.01 and a plot of log likelihood scores with generation), we culled a burn-in set of 10,000 trees and calculated the posterior probabilities. Trees were midpoint rooted.

For the phylogeny of yeasts, we used the five genes (Additional File 6) with the greatest number of strong reliability values from the list of the best 10 performing genes for recovering fungal phylogeny (as determined by a genome-scale analysis [45] for topological correctness). For fungi, orthologous nuclear genes with strong support values for a given node (bootstrap > 90 and posterior probabilities > 0.95) rarely disagree [46,47]. We chose species from FUNYBASE [48] that had all of the genes of interest, and added genomic sequences from an additional species of *Schizosaccharomyces*, and from three genomes where we detected totivirid-like sequences (Additional File 1; a fourth genome with totivirid sequences, *D. hansenii *was already part of FUNYBASE). Concatenated sequences were aligned in MAFFT [49] and then exposed to culling from GBlocks [50]. We carried out maximum likelihood and Bayesian analyses as for the totivirid alignments above.

A test of the homogeneity of substitution patterns between viral and fungal sequences for the Cp-like genes was carried out in MEGA4 [51]. We used the Disparity Index test [52] with *P*-values estimated from 1000 Monte Carlo based replicates (Additional File 2). The most divergent sequence (*S. cerevisiae *virus La (L-BC)) and the shortest sequence (*C. parapsilosis*) were culled for this analysis because we wanted to retain informative alignment positions when gapped sites are completely deleted. After positions with gaps and missing data were eliminated a dataset of 1446 positions was retained.

Tests of neutral evolution for the Cp-like gene copies in fungi were carried out in MEGA4. The test statistic is (*d*N - *d*S) where *d*S and *d*N are the numbers of synonymous and nonsynonymous substitutions per site, respectively. The variance of the difference was computed using the bootstrap method (500 replicates). Analyses were conducted using the Kumar method in MEGA4 [51]. Ka/Ks ratios were calculated by the Ka/Ks calculator http://services.cbu.uib.no/tools/kaks and plotted on a tree using the methods of [53]. GC content was estimated from the alignment. The ratio of nonsynonymous (Ka) to synonymous (Ks) nucleotide substitution rates is an indicator of selective pressures on genes. A ratio of less than one indicates selective pressure to conserve protein sequence. Note that values are averaged over sites.

## Abbreviations

Cp: capsid polypeptide; EST: expressed sequence tag; HGT: horizontal gene transfer; NIRV: non-retroviral integrated RNA virus; PCR: polymerase chain reaction; RdRp: RNA dependant RNA polymerase; RTPCR: reverse transcriptase PCR.

## Authors' contributions

DJT and JB conceived the study and co-wrote the paper. JB carried out dsRNA and viral particle isolations, designed primers and identified residues essential for capsid functioning. DJT carried out DNA sequence assembly, PCR, phylogenetics and evolutionary analyses.

## Supplementary Material

Additional file 1 Midpoint rooted maximum likelihood phylogram of yeast-like fungi based on a concatenation of the five single copy protein-coding genes identified as the most phylogenetically reliable in fungal genomes. Click here for file

Additional file 2 Test of the homogeneity of substitution patterns between fungal and viral copies of capsid-like protein nucleotide sequences. Click here for file

Additional file 3 Primers used for polymerase chain reaction (PCR) and reverse transcriptase-PCR of *Totivirus*-like regions of yeast genomes and exogenous *Totivirus*. Click here for file

Additional file 4 Viral and fungal sequences and accession numbers used for phylogenetic analysis of the RdRp-like regions of totivirids and *Totivirus*-like sequences in fungi. Click here for file

Additional file 5 Viral and fungal accession numbers used for phylogenetic analysis of the Cp-like regions of totivirids and *Totivirus*-like sequences in fungi. Click here for file

Additional file 6 Fungal genes and accession numbers used for phylogenetic analysis of the budding yeasts. Click here for file

## References

[B1] Long M Betran E Thornton K Wang W The origin of new genes: glimpses from the young and old Nat Rev Genet 2003 4 11 865 875 10.1038/nrg1204 14634634

[B2] Doolittle WF You are what you eat: a gene transfer ratchet could account for bacterial genes in eukaryotic nuclear genomes Trends Genet 1998 14 8 307 311 10.1016/S0168-9525(98)01494-2 9724962

[B3] Roy SW Gilbert W The evolution of spliceosomal introns: patterns, puzzles and progress Nat Rev Genet 2006 7 3 211 221 16485020 10.1038/nrg1807

[B4] Meyers BC Tingey SV Morgante M Abundance, distribution, and transcriptional activity of repetitive elements in the maize genome Genome Res 2001 11 10 1660 1676 10.1101/gr.188201 11591643 PMC311155

[B5] Kazazian HH Jr Mobile elements: drivers of genome evolution Science 2004 303 5664 1626 1632 10.1126/science.1089670 15016989

[B6] Dujon B Sherman D Fischer G Durrens P Casaregola S Lafontaine I De Montigny J Marck C Neuvéglise C Talla E Goffard N Frangeul L Aigle M Anthouard V Babour A Barbe V Barnay S Blanchin S Beckerich JM Beyne E Bleykasten C Boisramé A Boyer J Cattolico L Confanioleri F De Daruvar A Despons L Fabre E Fairhead C Ferry-Dumazet H Genome evolution in yeasts Nature 2004 430 6995 35 44 10.1038/nature02579 15229592

[B7] Dujon B Yeasts illustrate the molecular mechanisms of eukaryotic genome evolution Trends Genetics 2006 22 7 375 387 10.1016/j.tig.2006.05.007 16730849

[B8] Jeffries TW Grigoriev IV Grimwood J Laplaza JM Aerts A Salamov A Schmutz J Lindquist E Dehal P Shapiro H Jin YS Passoth V Richardson PM Genome sequence of the lignocellulose-bioconverting and xylose-fermenting yeast *Pichia stipitis* Nature Biotechnol 2007 25 3 319 326 10.1038/nbt1290 17334359

[B9] Butler G Rasmussen MD Lin MF Santos MA Sakthikumar S Munro CA Rheinbay E Grabherr M Forche A Reedy JL Evolution of pathogenicity and sexual reproduction in eight Candida genomes Nature 2009 459 7247 657 662 10.1038/nature08064 19465905 PMC2834264

[B10] Frank AC Wolfe KH Evolutionary capture of viral and plasmid DNA by yeast nuclear chromosomes Eukaryot Cell 2009 8 10 1521 1531 10.1128/EC.00110-09 19666779 PMC2756859

[B11] Flegel TW Hypothesis for heritable, anti-viral immunity in crustaceans and insects Biol Direct 2009 4 32 10.1186/1745-6150-4-32 19725947 PMC2757015

[B12] Holmes EC The Evolution and Emergence of RNA Viruses 2009 New York: Oxford University Press

[B13] Staginnus C Iskra-Caruana ML Lockhart B Hohn T Richert-Poggeler KR Suggestions for a nomenclature of endogenous pararetroviral sequences in plants Arch Virol 2009 154 7 1189 1193 10.1007/s00705-009-0412-y 19521659

[B14] Crochu S Cook S Attoui H Charrel RN De Chesse R Belhouchet M Lemasson JJ de Micco P de Lamballerie X Sequences of flavivirus-related RNA viruses persist in DNA form integrated in the genome of *Aedes spp*. mosquitoes J Gen Virol 2004 85 Pt 7 1971 1980 10.1099/vir.0.79850-0 15218182

[B15] Maori E Tanne E Sela I Reciprocal sequence exchange between non-retro viruses and hosts leading to the appearance of new host phenotypes Virology 2007 362 2 342 349 10.1016/j.virol.2006.11.038 17275871

[B16] Tanne E Sela I Occurrence of a DNA sequence of a non-retro RNA virus in a host plant genome and its expression: evidence for recombination between viral and host RNAs Virology 2005 332 2 614 622 10.1016/j.virol.2004.11.007 15680426

[B17] Geuking MB Weber J Dewannieux M Gorelik E Heidmann T Hengartner H Zinkernagel RM Hangartner L Recombination of retrotransposon and exogenous RNA virus results in nonretroviral cDNA integration Science 2009 323 5912 393 396 10.1126/science.1167375 19150848

[B18] Keeling PJ Palmer JD Horizontal gene transfer in eukaryotic evolution Nat Rev Genet 2008 9 8 605 618 10.1038/nrg2386 18591983

[B19] Ragan MA Harlow TJ Beiko RG Do different surrogate methods detect lateral genetic transfer events of different relative ages? Trends Microbiol 2006 14 1 4 8 10.1016/j.tim.2005.11.004 16356716

[B20] Ragan MA On surrogate methods for detecting lateral gene transfer FEMS Microbiol Lett 2001 201 2 187 191 10.1111/j.1574-6968.2001.tb10755.x 11470360

[B21] Stanhope MJ Lupas A Italia MJ Koretke KK Volker C Brown JR Phylogenetic analyses do not support horizontal gene transfers from bacteria to vertebrates Nature 2001 411 6840 940 944 10.1038/35082058 11418856

[B22] Bertsch C Beuve M Dolja VV Wirth M Pelsy F Herrbach E Lemaire O Retention of the virus-derived sequences in the nuclear genome of grapevine as a potential pathway to virus resistance Biol Direct 2009 4 21 10.1186/1745-6150-4-21 19558678 PMC2714080

[B23] Bruenn J Mahy BWJ, van Regenmortel MHV The Ustilago maydis viruses Encyclopedia of Virology 2008 5 3 Amsterdam: Elsevier 214 219 full_text

[B24] Nikoh N Nakabachi A Aphids acquired symbiotic genes via lateral gene transfer BMC Biol 2009 7 12 10.1186/1741-7007-7-12 19284544 PMC2662799

[B25] Naitow H Tang J Canady M Wickner RB Johnson JE L-A virus at 3.4 A resolution reveals particle architecture and mRNA decapping mechanism Nat Struct Biol 2002 9 10 725 728 10.1038/nsb844 12244300

[B26] Tang J Naitow H Gardner NA Kolesar A Tang L Wickner RB Johnson JE The structural basis of recognition and removal of cellular mRNA 7-methyl G 'caps' by a viral capsid protein: a unique viral response to host defense J Mol Recognit 2005 18 2 158 168 10.1002/jmr.724 15597333

[B27] Ghabrial SA Origin, adaptation and evolutionary pathways of fungal viruses Virus Genes 1998 16 1 119 131 10.1023/A:1007966229595 9562896 PMC7089520

[B28] Hastie ND Brennan V Bruenn J No homology between double-stranded RNA and nuclear DNA of yeast J Virol 1978 28 1002 1005 366175 10.1128/jvi.28.3.1002-1005.1978 PMC525824

[B29] Ghabrial S BWJ Mahy, van Regenmortel MHV Totiviruses Encyclopedia of Virology 2008 5 3 Academic Press 163 174 full_text

[B30] Diamond ME Dowhanick JJ Nemeroff ME Pietras DF Tu C-L Bruenn JA Overlapping genes in a yeast dsRNA virus J Virol 1989 63 3983 3990 2668562 10.1128/jvi.63.9.3983-3990.1989 PMC250995

[B31] Tzeng T-H Tu C-L Bruenn JA Ribosomal frameshifting requires a pseudoknot in the yeast double-stranded RNA virus J Virol 1992 66 2 999 1006 1731118 10.1128/jvi.66.2.999-1006.1992 PMC240802

[B32] Kang J Wu J Bruenn JA Park C The H1 double-stranded RNA genome of Ustilago maydis virus-H1 encodes a polyprotein that contains structural motifs for capsid polypeptide, papain-like protease, and RNA-dependent RNA polymerase Virus Res 2001 76 2 183 189 10.1016/S0168-1702(01)00250-7 11410317

[B33] Taylor JW Heitman J, Filler SG, Edwards JE Jr, Mitchell AP Evolution of human-pathogenic fungi: phylogenies and species Molecular Principles of Fungal Pathogenesis 2006 Washington D.C.: ASM press 113 132

[B34] Jern P Coffin JM Effects of retroviruses on host genome function Annu Rev Genet 2008 42 709 732 10.1146/annurev.genet.42.110807.091501 18694346

[B35] Lesage P Todeschini AL Happy together: the life and times of Ty retrotransposons and their hosts Cytogenet Genome Res 2005 110 1-4 70 90 10.1159/000084940 16093660

[B36] Valle RP Wickner RB Elimination of L-A double-stranded RNA virus of *Saccharomyces cerevisiae *by expression of *gag *and *gag-pol *from L-A cDNA clone J Virol 1993 67 5 2764 2771 8474174 10.1128/jvi.67.5.2764-2771.1993 PMC237600

[B37] Yao W Bruenn JA Interference with replication of two double-stranded RNA viruses by production of N-terminal fragments of capsid polypeptides Virology 1995 214 215 221 10.1006/viro.1995.9938 8525618

[B38] Reimann-Philipp U Mechanisms of resistance: expression of coat protein Methods Mol Biol 1998 81 521 532 9760540 10.1385/0-89603-385-6:521

[B39] Holm CA Oliver SG Newman AM Holland LE McLaughlin CS Wagner EK Warner RC The molecular weight of yeast P1 double-stranded RNA J Biol Chem 1978 253 8332 8336 361739

[B40] Franklin RM Purification and properties of the replicative intermediate of the RNA bacteriophage R17 Proc Natl Acad Sci USA 1966 55 1504 1511 10.1073/pnas.55.6.1504 5227669 PMC224351

[B41] Naitow H Canady MA Lin T Wickner RB Johnson JE Purification, crystallization, and preliminary X-ray analysis of L-A: a dsRNA yeast virus J Struct Biol 2001 135 1 1 7 10.1006/jsbi.2001.4371 11562160

[B42] Loytynoja A Goldman N Phylogeny-aware gap placement prevents errors in sequence alignment and evolutionary analysis Science 2008 320 5883 1632 1635 10.1126/science.1158395 18566285

[B43] Stamatakis A Hoover P Rougemont J A rapid bootstrap algorithm for the RAxML Web servers Syst Biol 2008 57 5 758 771 10.1080/10635150802429642 18853362

[B44] Ronquist F Huelsenbeck JP MrBayes 3: Bayesian phylogenetic inference under mixed models Bioinformatics 2003 19 12 1572 1574 10.1093/bioinformatics/btg180 12912839

[B45] Aguileta G Marthey S Chiapello H Lebrun MH Rodolphe F Fournier E Gendrault-Jacquemard A Giraud T Assessing the performance of single-copy genes for recovering robust phylogenies Syst Biol 2008 57 4 613 627 10.1080/10635150802306527 18709599

[B46] Taylor DJ Piel WH An assessment of accuracy, error, and conflict with support values from genome-scale phylogenetic data Mol Biol Evol 2004 21 8 1534 1537 10.1093/molbev/msh156 15140947

[B47] Rasmussen MD Kellis M Accurate gene-tree reconstruction by learning gene- and species-specific substitution rates across multiple complete genomes Genome Res 2007 17 12 1932 1942 10.1101/gr.7105007 17989260 PMC2099600

[B48] Marthey S Aguileta G Rodolphe F Gendrault A Giraud T Fournier E Lopez-Villavicencio M Gautier A Lebrun MH Chiapello H FUNYBASE: a FUNgal phYlogenomic dataBASE Bmc Bioinformatics 2008 9 456 10.1186/1471-2105-9-456 18954438 PMC2600828

[B49] Katoh K Asimenos G Toh H Multiple alignment of DNA sequences with MAFFT Methods Mol Biol 2009 537 39 64 full_text 19378139 10.1007/978-1-59745-251-9_3

[B50] Talavera G Castresana J Improvement of phylogenies after removing divergent and ambiguously aligned blocks from protein sequence alignments Syst Biol 2007 56 4 564 577 10.1080/10635150701472164 17654362

[B51] Tamura K Dudley J Nei M Kumar S MEGA4: Molecular evolutionary genetics analysis (MEGA) software version 4.0 Mol Biol Evol 2007 24 8 1596 1599 10.1093/molbev/msm092 17488738

[B52] Kumar S Gadagkar SR Disparity index: a simple statistic to measure and test the homogeneity of substitution patterns between molecular sequences Genetics 2001 158 3 1321 1327 11454778 10.1093/genetics/158.3.1321 PMC1461708

[B53] Liberles DA Evaluation of methods for determination of a reconstructed history of gene sequence evolution Mol Biol Evol 2001 18 11 2040 2047 11606700 10.1093/oxfordjournals.molbev.a003745

