# Supplementary figures and images for: The evolution of novel fungal genes from non-retroviral RNA viruses
Source: BMC Biol. 2009 Dec 18;7:88. doi: 10.1186/1741-7007-7-88 (PMC2805616; doi:10.1186/1741-7007-7-88)

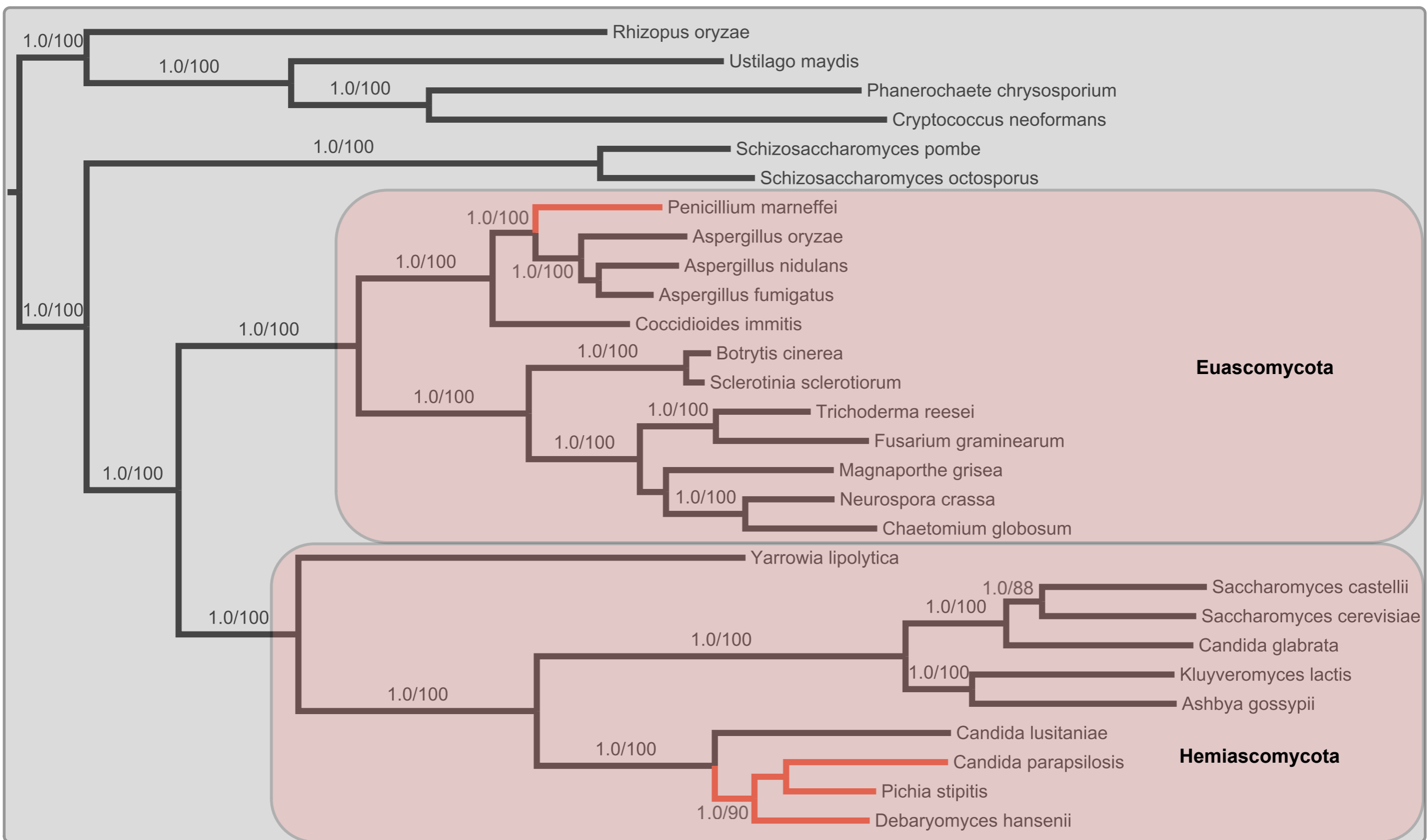

0.1

Supplement: Additional file 1 — Midpoint rooted maximum likelihood phylogram of yeast-like fungi based on a concatenation of the five single copy protein-coding genes identified as the most phylogenetically reliable in fungal genomes. [file 1741-7007-7-88-S1.PDF]
