# Supplementary material for: The evolution of novel fungal genes from non-retroviral RNA viruses
Source: BMC Biol. 2009 Dec 18;7:88. doi: 10.1186/1741-7007-7-88 (PMC2805616; doi:10.1186/1741-7007-7-88)
Supplement: Additional file 2 — Test of the homogeneity of substitution patterns between fungal and viral copies of capsid-like protein nucleotide sequences. [file 1741-7007-7-88-S2.DOC]

Additional file 2. Test of the homogeneity of substitution patterns between fungal and viral copies of Capsid-like protein nucleotide sequences. The Disparity Index is shown where values greater than 0 indicate larger differences in base composition than expected by evolutionary divergence. A Monte Carlo test (1000 replicates) was used to estimate the P-values, which are significant (P<0.05) for values shown in red. Contrasts between viral and fungal sequences are shown in yellow and reveal substitution pattern differences not due to chance or to evolutionary distance. Note that the only fungal gene (*P. stipitis* Cp1) to differ from other fungal genes in the Disparity index, is not significantly different from its presumed ancestral fungal gene copy (*P. stipitis* Cp3). The key to the cell numbers is given below the table.

|  | 1 | 2 | 3 | 4 | 5 | 6 | 7 | 8 | 9 |
| --- | --- | --- | --- | --- | --- | --- | --- | --- | --- |
| 1 |  |  |  |  |  |  |  |  |  |
| 2 | 0.000 |  |  |  |  |  |  |  |  |
| 3 | 1.242 | 3.297 |  |  |  |  |  |  |  |
| 4 | 1.125 | 3.013 | 0.004 |  |  |  |  |  |  |
| 5 | 3.062 | 5.587 | 0.173 | 0.183 |  |  |  |  |  |
| 6 | 1.421 | 3.720 | 0.000 | 0.000 | 0.104 |  |  |  |  |
| 7 | 3.171 | 6.198 | 0.000 | 0.204 | 0.000 | 0.718 |  |  |  |
| 8 | 2.148 | 4.103 | 0.156 | 0.219 | 0.024 | 0.581 | 0.564 |  |  |
| 9 | 4.216 | 6.031 | 2.186 | 2.446 | 1.878 | 3.320 | 2.481 | 0.259 |  |

| 1. *Saccharomyces cerevisiae* virus La (L-BC) |
| --- |
| 2. Black raspberry virus F |
| 3. *Debaryomyces hansenii* Cp1 |
| 4. *D. hansenii* Cp2 |
| 5. *Penicillium marneffei* |
| 6. *Pichia stipitis* Cp4 |
| 7. *P. stipitis* Cp2 |
| 8. *P. stipitis* Cp3 |
| 9. *P. stipitis* Cp1 |
