# Supplementary material for: The evolution of novel fungal genes from non-retroviral RNA viruses
Source: BMC Biol. 2009 Dec 18;7:88. doi: 10.1186/1741-7007-7-88 (PMC2805616; doi:10.1186/1741-7007-7-88)
Supplement: Additional file 3 — Primers used for polymerase chain reaction (PCR) and reverse transcriptase-PCR of Totivirus-like regions of yeast genomes and exogenous Totivirus. [file 1741-7007-7-88-S3.DOC]

**Additional file 3.** **Primers used for PCR and RT-PCR of *Totivirus*-like regions of yeast genomes and exogenous *Totivirus.*** Underlined regions indicate 5’ linker sequence. Chromosomal locations and matching PCR primers are provided.

| **Target species** | **Primer sequence** | **Genomic location** | **Primer name (PCR primer pairing)** |
| --- | --- | --- | --- |
| *Pichia stipitis* | 5’-AGAGAAAGTAGGAGAGCCATTTCTGCAGG-3’ | CHR 7: 500115-500143 | PsCp2F (PsIGR) |
| *P. stipitis* | 5’-AGTCTGTACGTACACTTCTCATTTAAGTGG-3’ | CHR 7: 500706-500735 | PsIGF (PsRdRpR) |
| *P. stipitis* | 5’-CCACTTAAATGAGAAGTGTACGTACAGACT-3’ | CHR 7: 500735-500706 | PsIGR (PsCp2F) |
| *P. stipitis* | 5’-GAAATAGTTCGGACCAATTGCGCGCGTTGC-3’ | CHR 7: 501155-501126 | PsRdRpR (PsIGF) |
| *P. stipitis* | 5’- CCGCAGGTCTCAAATGTGTA -3’ | CHR 7: 503100- 503119 | PsRdRpF2 (PsRdRpR2) |
| *P. stipitis* | 5’- TTGAGTGCGACACTAAAGCACAAACCCG -3’ | CHR 7: 503390-503417 | PsRdRpF (PsCp3R) |
| *P. stipitis* | 5’- TACGAGGATTCAGGCAGTCC -3’ | CHR 7: 503503- 503484 | PsRdRpR2 (PsRdRpF2) |
| *P. stipitis* | 5’- CCCGTCGATTTCAGTCTACGTTCTATC -3’ | CHR 7: 503868- 503842 | PsCp3R (PsRdRpF) |
| *Debaryomyces* *hansenii* | 5’-GTGTAGTCCTGGGAGGCAGTCAAAGTACGAG-3’ | CHR B: 1022629-1022659 | DhCp1F (DhIGR) |
| *D.* *hansenii* | 5’-CCAAGTAGAAGTGTATGCTTAACAGCATAAG-3’ | CHR B: 1023843-1023873 | DhIGF  (DhRdRpR) |
| *D.* *hansenii* | 5’-CTTATGCTGTTAAGCATACACTTCTACTTGG-3’ | CHR B: 1023873-1023843 | DhIGR (DhCp1F) |
| *D.* *hansenii* | 5’-ATGAGCAGACTGATAAAAGTCTCTATTGATAC-3’ | CHR B: 1024716 -1024685 | DhRdRpR (DhIGF) |
| *D.* *hansenii* | 5’-CTGGAACCCACTCCCATCTA-3’ | CHR B: 1025976-1025995 | DhRdRpF2 (DhRdRpR2) |
| *D.* *hansenii* | 5’-TACGGTTTCCTCCTTCATGG-3’ | CHR B:1026373-  1026354 | DhRdRpR2 (DhRdRpF2) |
| *D.* *hansenii* | 5’-ACATGCATGC CAATTAGCTCTACTCTTGACCAGC-3’ | CHR B:1026951-  1026974 | DhRdRpF (DhCp2R) |
| *D.* *hansenii* | 5’- ACATGCATGC TGCAACTAATATTGTTGCTCAAGTTATGTTG -3’ | CHR B: 1030734-  1030764 | DhCp2R (DhRdRpF) |
| *Saccharomyces cerevisiae* virus L1 (LA) | 5’- GTGCAGGCTGGTGCGCATTCAGTGTATC -3’ | 1821-1848 | ScvCpF (ScvRdRpR) |
| *S. cerevisiae* virus L1 (LA) | 5’- TCTGTACGTACAAGGCATAAGTGTCACTG -3’ | 2339-2311 | ScvRdRpR (ScvCpF) |
