# Supplementary material for: The evolution of novel fungal genes from non-retroviral RNA viruses
Source: BMC Biol. 2009 Dec 18;7:88. doi: 10.1186/1741-7007-7-88 (PMC2805616; doi:10.1186/1741-7007-7-88)
Supplement: Additional file 4 — Viral and fungal sequences and accession numbers used for phylogenetic analysis of the RdRp-like regions of totivirids and Totivirus-like sequences in fungi. [file 1741-7007-7-88-S4.DOC]

Additional file 4. Viral and fungal sequences and Accession numbers used for phylogenetic analysis of the RdRp-like regions of totivirids and *Totivirus*-like sequences in fungi.

| **Species** | **Genbank Accession** |
| --- | --- |
| Amasya cherry disease-associated mycovirus 4 | CAJ29959 |
| Amasya cherry disease-associated mycovirus 1 | AM085134 |
| Saccharomyces cerevisiae virus L1 (L-A) | AAA50321 |
| Uromyces appendiculatus EST | FE660516.1, FE660577.1 |
| Pichia stipitis | NC_009047 |
| Penicillium marneffei | ABAR01000272 |
| Debaryomyces hansenii | CR382134 |
| Saccharomyces cerevisiae virus La (L-BC) | NP_042581.1 |
| Black raspberry virus F | ABU55399 |
| Ustilago maydis virus H1 | NP_620728 |
| Trichomonas vaginalis virus 3 | NP_659390 |
| Trichomonas vaginalis virus 1 | NP_624323 |
| Trichomonas vaginalis virus IH2 | ABC86751 |
| Trichomonas vaginalis virus T1 | NP_620730 |
| Trichomonas vaginalis virus Changchun isolate | DQ528812 |
| Leishmaniavirus 1-1 | NP_041191 |
| Leishmaniavirus 1-4 | NP_619653 |
| Leishmaniavirus 2-1 | NP_043465 |
| Thielaviopsis basicola dsRNA virus 1 | YP_024728 |
| Botryotinia fuckeliana totivirus 1 | YP_001109580 |
| Helminthosporium victoriae virus 190S | NP_619670.1 |
| Sphaeropsis sapinea RNA virus 1 | NP_047558 |
| Magnaporthe oryzae virus 1 | YP_122352 |
| Ophiostoma minus totivirus | CAJ34336 |
| Helicobasidium mompa No.17 virus | NP_898833 |
| Epichloe festucae virus 1 | CAK02788 |
| Coniothyrium minitans virus | YP_392467 |
| Magnaporthe oryzae virus 2 | YP_001649206 |
| Gremmeniella abietina RNA virus L1 | NP_624332.2 |
| Gremmeniella abietina RNA virus L2 | YP_044807 |
| Aspergillus mycovirus 178 | ABX79995 |
| Sphaeropsis sapinea RNA virus 2 | NP_047560.1 |
| Eimeria brunetti RNA virus 1 | NP_108651 |
