# Supplementary material for: The evolution of novel fungal genes from non-retroviral RNA viruses
Source: BMC Biol. 2009 Dec 18;7:88. doi: 10.1186/1741-7007-7-88 (PMC2805616; doi:10.1186/1741-7007-7-88)
Supplement: Additional file 5 — Viral and fungal accession numbers used for phylogenetic analysis of the Cp-like regions of totivirids and Totivirus-like sequences in fungi. [file 1741-7007-7-88-S5.DOC]

Additional file 5. Viral and fungal Accession numbers used for phylogenetic analysis of the Cp-like regions of totivirids and *Totivirus*-like sequences in fungi. The gene notation is from Fig.1.

| **Species** | **Gene** | **Genbank Accession** |
| --- | --- | --- |
| *Pichia stipitis* | Cp3 | CP000501 |
| *Pichia stipitis* | Cp2 | XM_001386078 |
| *Pichia stipitis* | Cp1 | XM_001386077.1 |
| *Pichia stipitis* | Cp4 | CP000501 |
| *Candida parapsilosis* | Cp | CABE01000013 |
| *Debaryomyces hansenii* | Cp1 | XP_457517 |
| *Debaryomyces hansenii* | Cp2 | CR382134 |
| *Penicillium marneffei* | Cp | ABAR01000142.1 |
| *Saccharomyces cerevisiae* virus La (L-BC) | Cp | NP_042580.1 |
| *Saccharomyces cerevisiae* virus L1 (L-A) | Cp | AAA50320.1 |
| Black raspberry virus F | Cp | ABU55398.1 |
