# Supplementary material for: The evolution of novel fungal genes from non-retroviral RNA viruses
Source: BMC Biol. 2009 Dec 18;7:88. doi: 10.1186/1741-7007-7-88 (PMC2805616; doi:10.1186/1741-7007-7-88)
Supplement: Additional file 6 — Fungal genes and accession numbers used for phylogenetic analysis of the budding yeasts. [file 1741-7007-7-88-S6.DOC]

Additional file 6. Fungal genes and Accession numbers used for phylogenetic analysis of the budding yeasts (Additional file 1). Gene names are from FUNYBASE (http://genome.jouy.inra.fr/funybase/funybase_start.cgi). The sequence database numbers are either from FUNYBASE or from Genbank.

| **Species** | **FG533** | **MS456** | **MS378** | **FG570** | **FG595** |
| --- | --- | --- | --- | --- | --- |
| *Ashbya gossypii* | lclAGR201C | lclADR041W | lclADR331C | lclAGL040C | lclAEL305C |
| *Aspergillus fumigatus* | XP_754018.1 | XP_755347.1 | XP_749386.1 | XP_749729.1 | XP_754588.1 |
| *Aspergillus nidulans* | AN2294 | AN5992 | AN4639 | AN7451 | AN4956 |
| *Aspergillus oryzae* | XP_001817067.1 | AO090011000622 | AO090026000590 | AO090001000717 | AO090166000076 |
| *Botrytis cinerea* | BC1G_05075.1 | BC1G_05231.1 | BC1G_12846.1 | BC1G_15454.1 | BC1G_04829.1 |
| *Candida glabrata* | XP_449624.1 | XP_449582.1 | XP_449926.1 | XP_446609.1 | XP_448375.1 |
| *Candida lusitaniae* | CLUG_02018.1 | CLUG_04163.1 | CLUG_03364.1 | CLUG_05473.1 | CLUG_02660.1 |
| *Candida parapsilosis* | CABE01000008 | CABE01000024:1027081-1029459 | CABE01000015 | CABE01000015 | CABE01000024:1405039-1406847 |
| *Chaetomium globosum* | AAFU01000685 | CHG01076.1 | CHG06257.1 | CHG03059.1 | CHG05016.1 |
| *Coccidioides immitis* | CIMG_06045.2 | CIMG_07549.2 | CIMG_01822.2 | CIMG_00981.2 | CIMG_03584.2 |
| *Cryptococcus neoformans* | XP_567783.1 | XP_571487.1 | XP_570461.1 | XP_570379.1 | XP_566644.1 |
| *Debaryomyces hansenii* | XP_459174.1 | XP_457383.1 | XP_459649.1 | XP_458121.1 | XP_461633.1 |
| *Fusarium graminearum* | FG02040.1 | FG07105.1 | FG08704.1 | FG04117.1 | FG01086.1 |
| *Kluyveromyces lactis* | XP_455030.1 | XP_454998.1 | XP_451414.1 | XP_453440.1 | XP_452091.1 |
| *Magnaporthe grisea* | MGG_05481.5 | MGG_09300.5 | MGG_02874.5 | MGG_05247.5 | MGG_06868.5 |
| *Neurospora crassa* | NCU01229.2 | NCU08119.2 | NCU00621.2 | NCU00461.2 | NCU07982.2 |
| *Penicillium marneffei* | ABAR01000042 | ABAR01000008.1 | ABAR01000017.1 | ABAR01000002 | ABAR01000053.1 |
| *Phanerochaete chrysosporium* | jgiPhchr1565fgenesh1_pg.C_s | jgiPhchr1132425e_gww2.1.162 | jgiPhchr1135560e_gww2.13.9. | jgiPhchr18571fgenesh1_pg.C_ | jgiPhchr1192fgenesh1_pg.C_s |
| *Pichia stipitis* | XP_001382617.2 | XP_001384179 | XP_001387441.1 | XP_001384885.1 | XP_001384614.2 |
| *Rhizopus oryzae* | RO3G_13888.1 | RO3G_11608.1 | RO3G_00347.1 | RO3G_08360.1 | RO3G_03066.1 |
| *Saccharomyces castellii* | Scas_Contig721.20 | Scas_Contig721.36 | Scas_Contig684.14 | Scas_Contig677.17 | Scas_Contig717.73 |
| *Saccharomyces cerevisiae* | NP_015239.1 | NP_009761.1 | NP_012056.1 | NP_010066.1 | NP_013826.1 |
| *Schizosaccharomyces octosporus* | ABHY02000001 | ABHY02000002.1 | ABHY02000006.1 | ABHY02000002.1 | ABHY02000009.1 |
| *Schizosaccharomyces pombe* | NP_594862.1 | NP_596545.1 | NP_593370.1 | NP_588149.1 | NP_595382.1 |
| *Sclerotinia sclerotiorum* | SS1G_07525.1 | SS1G_12751.1 | SS1G_09434.1 | SS1G_00002.1 | SS1G_06550.1 |
| *Trichoderma reesei* | 30746 | 33863 | 18640 | 44156 | 9089 |
| *Ustilago maydis* | UM00259.1 | UM06402.1 | UM00801.1 | UM05984.1 | UM03239.1 |
| *Yarrowia lipolytica* | XP_500809.1 | XP_501070.1 | XP_503648.1 | XP_503741.1 | XP_501277.1 |
